# Multiprofessional teamwork in Finnish health and social service centers, experiences of managers

**DOI:** 10.1093/eurpub/ckac131.376

**Published:** 2022-10-25

**Authors:** T Sinervo, L Hietapakka

**Affiliations:** Welfare State and Reform, Finnish Institute for Health and Welfare, Helsinki, Finland

## Abstract

**Background:**

Health and social service centers are developed for the first contact points in Finland. Long waiting times, shortage of staff and lack of coordination have been major problems in primary care. As a solution, multiprofessional teams and better collaboration between professionals have been built. This study explores, how the teams work and what challenges they have.

**Methods:**

The study is based on 16 interviews (25 managers) in 5 health and social services centers. County provided specialized care in the whole area as well as primary services in two centers, one center was provided by a municipality, and two by a private firm. The managers were asked about work organization, their experiences of the team model and about well-being of employees. The interviews were analyzed using content analysis.

**Results:**

The managers saw the team work as functioning rather well. They also highlighted effective digital services and task shifting. Easy consultation of GP’ was important as nurses were the first contact for clients in phone. In this call, the client’ case was tried to be handled as far as possible. The competences of new professionals, such as social workers or psychiatric nurses had high value for the team. But the service processes and team-building were in progress. Working in mutual facilities and easy consultation of professionals was seen important. In most organizations clients were separated to different teams based on their service needs (acute or chronic conditions), or earlier clientele. As a result, some teams gained a more burdensome clientele, which increased stress.

**Conclusions:**

Multiprofessional work can streamline and improve the treatment process, but this requires more information which ways to collaborate work best for the clients and professionals.

**Key messages:**

• Multiprofessional team work, task shifting and digital services may increase efficiency and well-being of workers at health and social centers.

• Easy consultation of GP’s is required for task shifting.

